# The Nathan Lewis Hatfield Prize for Original Research in Medicine

**Published:** 1905-03

**Authors:** 


### THE NATHAN LEWIS HATFIELD PRIZE FOR ORIGINAL RESEARCH IN MEDICINE.

Five hundred dollars will be awarded by the College of Physicians of Philadelphia to the author of the best essay submitted in competition on or before March 1, 1906, subject, "The Clinical and Pathological Diagnosis of Sarcoma." Essays must be typewritten, designated by a motto or device, and accompanied by a sealed envelope bearing the same motto or device, and containing the name and address of the author. They must embody original observations and researches. The Committee reserves the right to make no award if none of the essays submitted is considered worthy of the prize. Further information may be obtained by addressing Dr. Francis R. Packard, College of Physicians, 219 South 13th street, Philadelphia, Pa.—*Medical Record.*

